# Anti-Inflammatory Potential of 3-Hydroxy-β-Ionone from *Moringa oleifera:* Decreased Transendothelial Migration of Monocytes Through an Inflamed Human Endothelial Cell Monolayer by Inhibiting the IκB-α/NF-κB Signaling Pathway

**DOI:** 10.3390/molecules29245873

**Published:** 2024-12-12

**Authors:** Thitiya Luetragoon, Krai Daowtak, Yordhathai Thongsri, Pachuen Potup, Philip C. Calder, Kanchana Usuwanthim

**Affiliations:** 1 Department of Medical Technology, Faculty of Allied Health Sciences, Nakhon Ratchasima College, Nakhon Ratchasima 30000, Thailand; nok.hong.yok49@gmail.com; 2 Cellular and Molecular Immunology Research Unit, Faculty of Allied Health Sciences, Naresuan University, Phitsanulok 65000, Thailand; kraid@nu.ac.th (K.D.); yordhathai.k@gmail.com (Y.T.); pachuenp@nu.ac.th (P.P.); 3 School of Human Development and Health, Faculty of Medicine, University of Southampton, Southampton SO16 6YD, UK; p.c.calder@soton.ac.uk

**Keywords:** *Moringa oleifera* Lam., 3-hydroxy-β-ionone, endothelial cell, monocyte, anti-inflammation

## Abstract

Moringa leaves provide numerous health benefits due to their anti-inflammatory properties. This study presents the first evidence that endothelial cell inflammation can potentially be ameliorated by moringa leaf extract. Here, we established an experimental human blood vessel cell model of inflammation using EA.hy926 cells. TNF-α was added after pre-treating the cells with crude leaf extract from *Moringa oleifera* Lam., a constituent fraction of the extract, and the bioactive component 3-hydroxy-β-ionone. The extract and the active ingredient significantly decreased the levels of pro-inflammatory mediators such as IL-6, IL-8, and MCP-1; decreased IκB-α and NF-κB p65 phosphorylation; and decreased the expression of VCAM-1, PECAM-1, and ICAM-1, three significant adhesion molecules. Furthermore, they attenuated THP-1 monocyte adhesion to the EA.hy926 monolayer and decreased monocyte transmigration across the monolayer. These findings suggest that 3-hydroxy-β-ionone and moringa leaf extract have anti-inflammatory properties and can be used as therapeutic agents to reduce the progression of diseases involving the inflamed endothelium by decreasing the production of inflammatory cytokines, chemokines, and adhesion molecules. This is promising for conditions such as atherosclerosis and neuroinflammation.

## 1. Introduction

Endothelial cells (ECs) form the monolayer lining the lumen of blood and lymphatic vessels, creating a semi-permeable barrier limiting access to the underlying tissues. The endothelium acts as a dual-purpose interface, serving as a protective barrier separating blood from tissues and functioning as an endocrine organ [1]. ECs maintain blood fluidity and control regional blood flow. They are important for regulating vessel wall permeability, platelet adhesion and aggregation, and leukocyte activation [2].

Normal inflammation typically acts as a defensive mechanism that serves as a major initial line of resistance to microbes and in response to tissue injury. Mast cells, macrophages, and dendritic cells, which are resident cells of the innate immune system, are triggered by infection and injury, which causes them to produce cytokines and other agents that cause inflammation, such as interleukin (IL)-1β, IL-6, IL-8, and TNF-α, which activate nearby ECs [3]. ECs also produce these pro-inflammatory mediators, and a change in the inflammatory state results in thrombosis, altered vasomotor tone, and loss of barrier function [4].

Pro-inflammatory mediators also trigger endothelial cells to produce adhesion molecules such as intercellular adhesion molecule 1 (ICAM-1) and vascular cell adhesion molecule 1 (VCAM-1), as well as chemoattractants. Chemoattractants attract leukocytes such as neutrophils and monocytes to ECs, while adhesion molecules mediate the capture, rolling, arrest, and crawling of leukocytes on the luminal EC surface [5]. Leukocytes can subsequently migrate directly through the basement membrane and smooth muscle layer into the intima of the vessel wall. Several EC adhesion molecules and receptors, including junctional adhesion molecules (JAMs) and platelet endothelial cell adhesion molecule 1 (PECAM-1), participate in this transit across the endothelial barrier [5,6]. These actions induce leukocyte delivery to inflammatory sites [2].

Acute inflammatory reactions may progress to a chronic state if they are unable to eradicate the initiating trigger or if the healing process is hampered in some way. Although chronic inflammation can progress silently, it is considered a major contributor to several conditions including cardiovascular disease (CVD), neuroinflammation, diabetes, rheumatoid arthritis, and cancer [7,8]. CVD is the most common disease globally and has an increasing prevalence worldwide, as well as a substantial morbidity and mortality burden. The number of deaths from CVD has risen internationally, from 12.1 million in 1990 to 18.6 million in 2019 [9]. The main factor causing CVD (coronary artery disease, stroke, and peripheral vascular disease) is atherosclerosis [10]. Atherosclerosis is distinguished by the accumulation of lipids within the vessel wall, creation of a fibrous covering of smooth muscle cells and collagen, and infiltration of cells of the immune system such as macrophages, T cells, dendritic cells, and mast cells into the vessel wall [11,12]. This so-called plaque development can result in stenosis, a narrowing of the artery lumen that can lead to tissue infarction. The most frequent cause of acute coronary artery thrombosis, which results in myocardial infarction, is the rupture of atherosclerotic plaques [13,14].

Through regulating the adhesion and transendothelial trafficking of circulating leukocytes, vascular ECs are involved in the development of atherosclerosis and other disorders associated with inflammation [5]. Potential strategies for inhibiting the progression of atherosclerosis include reducing inflammatory responses in ECs and decreasing leukocyte transmigration. Recently, Baker et al. reported that eicosapentaenoic acid and docosahexaenoic acid from fatty fish can reduce the TNF-α-induced production of inflammatory mediators by EA.hy926 ECs and decrease the surface expression of ICAM-1 [15]. Another study demonstrated that andrographolide, a plant-derived terpenoid, significantly reduces TNF-α-induced ICAM-1 and monocyte adhesion in EA.hy926 cells by inhibiting the IκK/NF-κB signaling cascade [16].

The *Moringa oleifera* Lam. (MO) tree is common in tropical and subtropical nations, especially Thailand. MO leaves provide pharmacologically active extracts, chemicals, and traditional medicines [17]. This tree’s edible leaves, blossoms, fruits, and immature pods are found in many cultures’ traditional cuisines [18]. MO leaves are an excellent source of beneficial nutrients since they include high levels of protein, essential amino acids, omega-3 fatty acids, elements, including calcium, iron, and potassium, vitamins A, group B, C, and E, as well as a number of antioxidants and polyphenols [19,20]. MO leaves can also be used to make tea and other beverages and, as a powder, be incorporated in capsules to be used as a supplement [21]. Due to its nutrient profile, MO is considered to have high nutritional value [22].

According to research, MO leaves reduce complications by inhibiting the generation of nitric oxide and bacterial cell wall-induced cytokines that promote inflammation in RAW264.7 cells [23]. In a prior investigation, we discovered that MO leaves and their biologically active component 3-hydroxy-β-ionone (3-HBI) suppress inflammation via controlling the NF-κB pathway [24]. The eNOS-NO-sGC pathway is mostly activated by MO leaf extract, which decreases arterial blood pressure by causing small-resistance arteries to relax [25]. Despite these findings, the anti-inflammatory properties of MO leaf extract and its bioconstituents on ECs have been underexplored. Therefore, this study aimed to explore the influence of MO leaf extract and its bioactive constituent 3-HBI on cultured EA.hy926 ECs following stimulation with TNF-α. TNF-α was used as a stimulant because this cytokine plays an important role in the activation of NF-κB and the subsequent induction of adhesion molecules (ICAM-1, VCAM-1 and PECAM-1) and cytokines and chemokines in human ECs [15,26].

## 2. Results

### 2.1. Cytotoxicity of Moringa Leaf Extract and Its Bioactive Compound on EA.hy926 Cells

**Figure 1:**
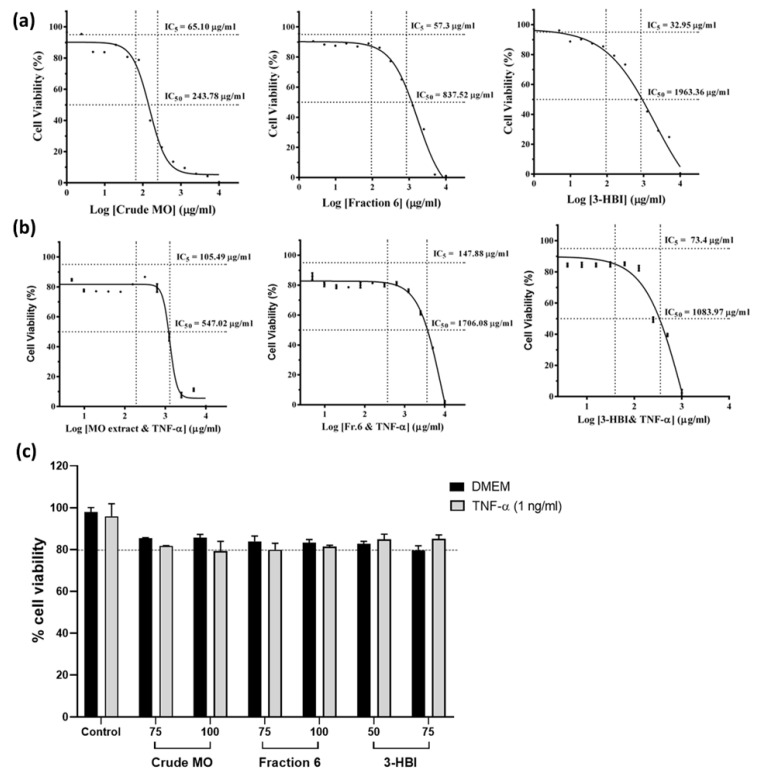
Effect of MO leaf extract, fraction 6, and 3-HBI on the viability of EA.hy926 cells determined using an MTT assay. (**a**) Sigmoidal curve fitting analysis of EA.hy926 cells after treatment for 24 h with MO extract, fraction 6, and 3-HBI. Black dots represent the percentage of cell viability at different doses of MO extract, Fr.6, and 3-HBI, displayed on a logarithmic scale. (**b**) followed by incubation with TNF-α (1 ng/mL) for 24 h. (**c**) Viability of EA.hy926 cells after incubation for 24 h with DMEM (control) or crude MO leaf extract (75, 100 µg/mL), fraction 6 (75, 100 µg/mL), and 3-HBI (50, 75 µg/mL), followed by incubation with or without TNF-α (1 ng/mL) for 24 h. The data are presented as the means ±SEM (*n* = 3).

The viability of EA.hy926 cells was examined using an MTT assay. Five percent (IC5) and half maximal (IC50) inhibitory concentrations were determined using sigmoidal curve fitting analyses and dose–response correlations. (Figure 1). The IC5 for crude MO extract, fraction 6 of the extract, and 3-HBI on the EA.hy926 cells was 65.1 µg/mL, 57.3 µg/mL, and 32.95 µg/mL, respectively (Figure 1a). The IC5 of crude MO extract, fraction 6, and 3-HBI after stimulation with TNF-α (1 ng/mL) for 24 h was 105.49 µg/mL, 147.88 µg/mL, and 73.4 µg/mL, respectively (Figure 1b). Cell viability after incubation with crude MO extract, fraction 6, and 3-HBI with or without stimulation with TNF-α was greater than 80%. Incubating the cells with TNF-α at 1 ng/mL for 24 h did not affect cell viability (Figure 1c).

### 2.2. Effects of the Bioactive Component in Moringa Leaf on Cytokine and Chemokine Production by EA.hy926 Cells

**Figure 2:**
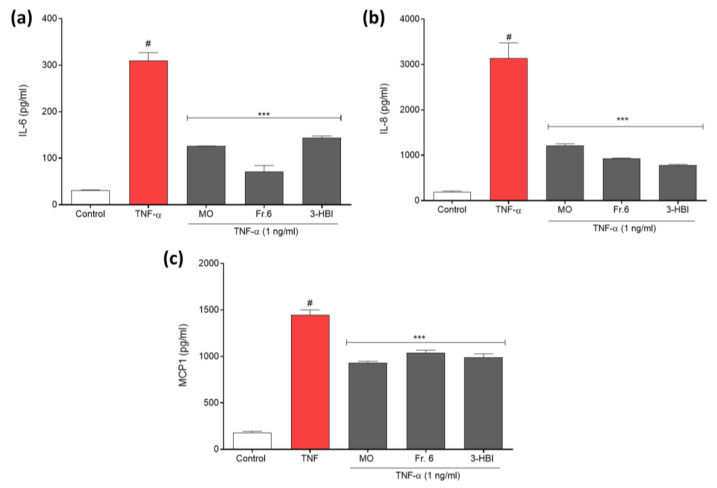
Effects of MO leaf extract, fraction 6, and 3-HBI on the levels of pro-inflammatory mediators in the supernatants of TNF-α-stimulated EA.hy926 endothelial cells. Concentrations of (**a**) IL-6, (**b**) IL-8, and (**c**) MCP-1 were measured in supernatants of EA.hy926 cells incubated for 24 h with DMEM (control; white bar), DMEM followed by 24 h of TNF-α stimulation (red bar), or with MO leaf extract, fraction 6, or 3-HBI followed by 24 h of TNF-α stimulation (gray bars). The data are presented as the means ±SEM (*n* = 3). # *p* < 0.001 compared to the control (no TNF-α). *** Statistically significant (*p* < 0.001) compared to TNF-α stimulation alone.

Pro-inflammatory mediators were measured by a Bio-Plex multiplex immunoassay system. Stimulation of EA.hy926 cells with 1 ng/mL TNF-α for 24 h resulted in a significant increase in the concentrations of all inflammatory mediators compared to those in unstimulated control cells cultured in DMEM. The IL-6, IL-8, and MCP-1 levels were significantly lower in the medium of the cells incubated with MO extract, fraction 6, or 3-HBI in comparison to the TNF-α-stimulated condition (Figure 2a–c). Thus, crude MO leaf extract and its constituent 3-HBI have anti-inflammatory potential.

### 2.3. Effect of the Bioactive Ingredient in Moringa Leaf Extract on ICAM-1 (CD54) and PECAM-1 (CD31) Expression in EA.hy926 Cells

**Figure 3:**
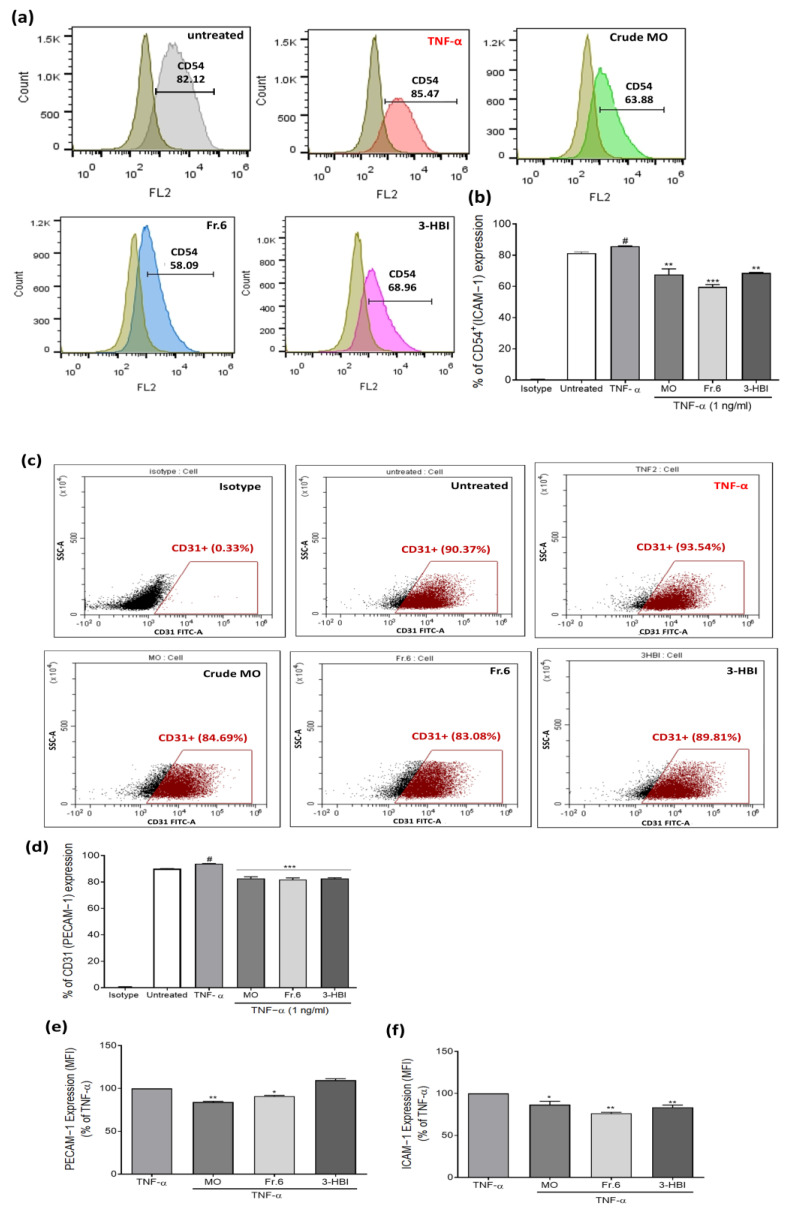
Flow cytometry analysis of adhesion molecules on the surface of EA.hy926 cells. (**a**) Histogram surface marker analysis, where the brown histograms represent the isotope control, while the light gray histogram indicates ICAM-1 (CD54) expression on untreated cells and cells treated with MO extract (green), Fr.6 (blue), and 3-HBI (pink). Bar graphs showing the percentages of (**b**) ICAM-1- and (**d**) PECAM-1-positive cells. (**c**) Dot plots representing endothelial cells gates within PECAM-1 (CD31)-positive cells. Bar graphs of the mean fluorescence intensity (MFI) of (**e**) PECAM-1 and (**f**) ICAM-1 expression. The data are presented as the means ±SEM (*n* = 3). # *p* < 0.001 compared to the isotype control. * Statistically significant (*p* < 0.05), ** *p* < 0.01, and *** *p* < 0.001 compared to TNF-α stimulation alone.

Following administration of crude moringa leaf extract, fraction 6, or 3-HBI for 24 h, EA.hy926 cells were triggered by TNF-α at 1 ng/mL for 6 h and then analyzed by flow cytometry. TNF-α increased the cell surface expression of ICAM-1 (85.47% positive cells) and PECAM-1 expression (93.54% positive cells) in the EA.hy926 cells. After culturing with crude MO extract, fraction 6 and 3-HBI, 63.88%, 58.09%, and 68.96% of the cells were ICAM-1-positive, while 84.69%, 83.08%, and 89.81% of the cells were PECAM-1-positive, respectively. Treatment with moringa extract, fraction 6, and 3-HBI significantly decreased the percentage of cells expressing ICAM-1 and PECAM-1 compared to the TNF-α-stimulated control cells (Figure 3a–d). Moreover, the crude extract, fraction 6, and 3-HBI treatments significantly downregulated the level of PECAM-1 and ICAM-1 expression in the positive cells (MFI) compared to TNF-α stimulation (Figure 3e,f). However, the 3-HBI treatment did not change the level of PECAM-1 expression (MFI).

### 2.4. Effects of the Bioactive Ingredient and Crude Moringa Leaf Extract on THP-1 Adherence to EA.hy926 Cells

**Figure 4:**
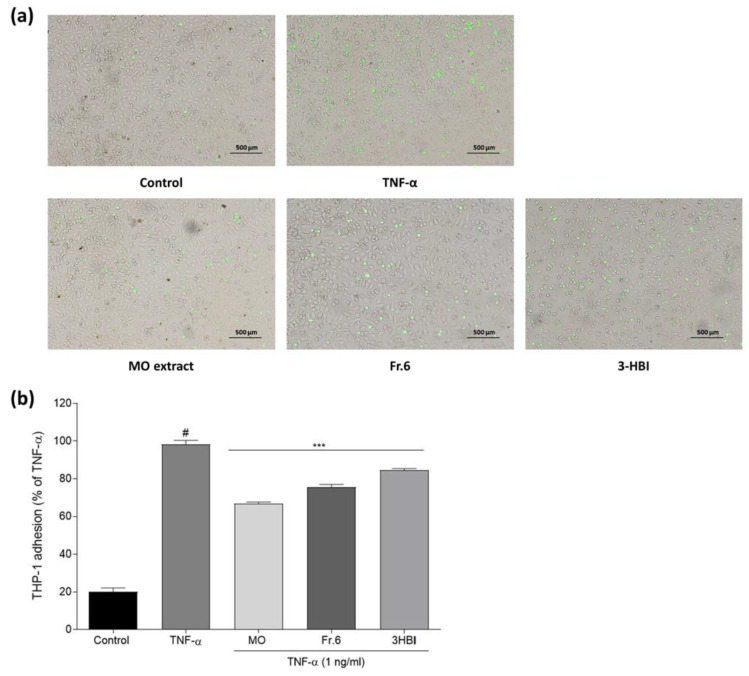
Adhesion of THP-1 monocytes to EA.hy926 cells incubated for 24 h with DMEM (control) or treatment with MO extract, fraction 6, and 3-HBI followed by stimulation with TNF-α (1 ng/mL) for 6 h and 1 h co-culture with calcein-labeled THP-1 cells. (**a**) Attached THP-1 cells were visualized by a Nikon H600l fluorescence microscope. (**b**) Cell adhesion was detected using a fluorescence plate reader at 480 nm/520 nm. THP-1 monocyte adhesion was measured as a percentage of TNF-α stimulated cells (TNF-α). The data are presented as the means ±SEM (*n* = 3). # Statistically significant (*p* < 0.001) compared to the control. *** Statistically significant (*p* < 0.001) compared to TNF-α.

To understand how crude MO leaf extract and its bioactive compound influence the interaction between monocytes and endothelial cells, we analyzed whether crude extract and 3-HBI could affect the adhesion of monocytic THP-1 cells to an endothelial cell monolayer. Figure 4a represents images of fluorescence-labeled THP-1 monocytes adhering to EA.hy926 cells with and without crude extract and 3-HBI exposure. Figure 4b shows that TNF-α-induced endothelial cell stimulation dramatically increased the adherence of calcein-labeled THP-1 cells to EA.hy926 cells when compared to unstimulated cells *(p* ≤ 0.001). Pre-treatment of TNF-α-stimulated EA.hy926 cells with crude extract, fraction 6, or 3-HBI significantly decreased calcein-labeled THP-1 adhesion compared to TNF-α-activated cells alone (*p* ≤ 0.001).

### 2.5. Effects of the Bioactive Ingredient and Crude Moringa Leaf Extract on the Movement of THP-1 Monocytes Through a Monolayer of Endothelial Cells

**Figure 5:**
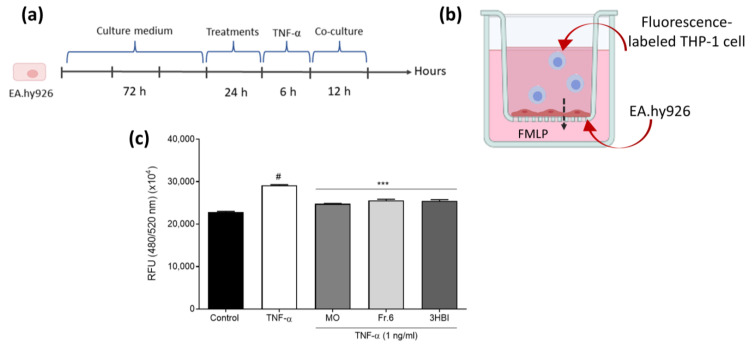
Analysis of THP-1 cell transmigration through an endothelial cell monolayer. EA.hy926 cells were cultured in an insert well for 72 h until the endothelial cells formed a monolayer. (**a**,**b**) Cells were incubated with DMEM (control) or with MO leaf extract, fraction 6, or 3-HBI for 24 h followed by stimulation with TNF-α (1 ng/mL) for 6 h and co-culture with calcein-AM-labeled THP-1 cells for 12 h at 37 °C. FMLP: N-Formylmethionyl-leucyl-phenylalanine. (**c**) The relative fluorescence unit (RFU) of the medium under the insert was measured using a fluorescence plate reader at Ex/Em = 480/520 nm in the end-point mode. The data are presented as the means ±SEM (*n* = 3). # *p* < 0.001 compared to the control. *** Statistically significant (*p* < 0.001) compared to TNF-α stimulation.

In order to investigate the effects of MO leaf extract and 3-HBI on monocyte migration, we analyzed the transmigration of monocytic THP-1 cells through an endothelial cell monolayer by a transwell transmigration assay. Endothelial cells were added to the insert until a monolayer was established. They were pre-treated with MO leaf extract, fraction 6, or 3-HBI and stimulated with TNF-α (Figure 5a). Then, the THP-1 monocytes were stained with fluorescent substrate (calcein AM) and placed in the upper chamber of the endothelial cell monolayer (Figure 5b). TNF-α stimulation of the endothelial cells significantly increased the migration of THP-1 monocytes compared to unstimulated controls (*p* < 0.001). THP-1 monocyte transmigration through the endothelial monolayer was significantly decreased after pre-treatment with crude MO extract, fraction 6, or 3-HBI followed by TNF-α stimulation (Figure 5c).

### 2.6. Effects of the Bioactive Ingredient and Crude Moringa Leaf Extract on ICAM-1, VCAM-1, Total NF-κB P65, pNF-κB P65, Total IκB-α, and pIκB-α Protein Expression in EA.hy926 Cells

**Figure 6:**
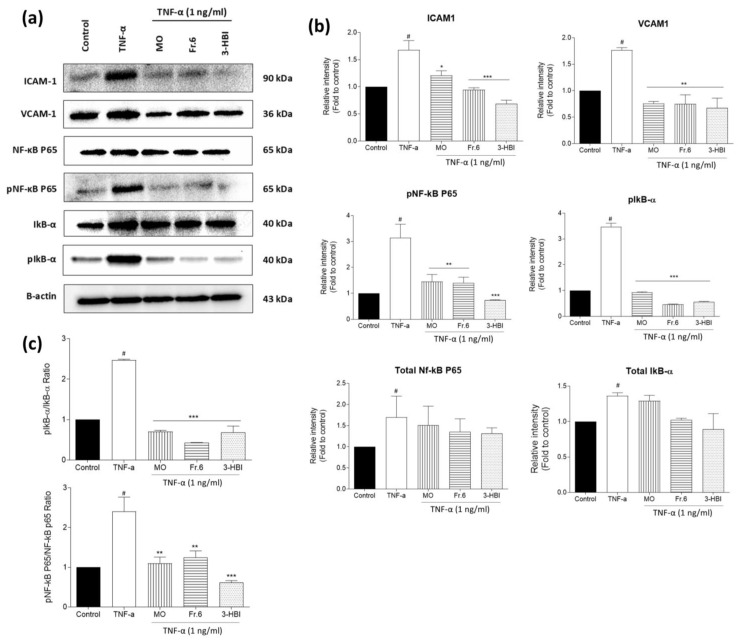
Effects of crude MO leaf extract, fraction 6, and 3-HBI on protein expression in EA.hy926 cells. (**a**) Band intensity of total protein levels of ICAM-1, VCAM-1, NF-κB P65, p-NF-κB P65, IkB-α, and p-IκB-α. β-actin was used as loading control. (**b**) Bar graphs represent the relative intensity of protein expression in control and treatment groups. (**c**) The ratio of phosphorylated NF-κB P65 and IκB-α to total protein levels of NF-κB P65 and IκB-α. Endothelial cells were exposed to crude MO extract (75 µg/mL), fraction 6 (75 µg/mL), and 3-HBI (50 µg/mL) for 24 h followed by stimulation with TNF-α (1 ng/mL) for 6 h. The cell extracts were subjected to 10–12% SDS-PAGE prior to Western blot analysis. β-actin was used as an internal control. The data are presented as the means ± SEM (*n* = 3). # *p*< 0.001 compared to the control. Statistically significant compared to TNF-α stimulation alone: *p < 0.05, ** *p* < 0.01, and *** *p* < 0.001. All original blots are presented in Supplementary Figures S4–S11.

We next determined whether TNF-α-induced NF-κB activation and the downstream signaling cascade were inhibited by MO leaf extract and 3-HBI. TNF-α induced both NF-κB P65 and IκB-α phosphorylation, and the activation of NF-κB P65 and IκB-α was significantly attenuated by pre-treatment with crude moringa leaf extract and the active component (3-HBI) in the EA.hy926 cells (Figure 6a,b). Moringa leaf extract and its active compound substantially decreased ICAM-1 and VCAM-1 expression when compared to stimulation with TNF-α alone (*p* < 0.001) (Figure 6b). Pre-treatment with MO leaf extract, fraction 6, and 3-HBI significantly decreased the ratio of pNF-κB P65/NF-κB P65 and pIκB-α/IκB-α (Figure 6c). These results demonstrated that the reduction in adhesion molecule (ICAM-1 and VCAM-1) expression in EA.hy926 cells by MO extract and 3-HBI might be related to the suppression of the NF-κB pathway.

## 3. Discussion

An important arterial condition known as atherosclerosis is characterized by persistent inflammation that emerges as a result of the recruitment of leukocytes, particularly monocytes, into the subendothelial region, followed by the production of several cytokines and the transformation of monocytes into foam cells [27]. Through leukocyte transmigration into the intimal layer, production of adhesion molecules, and leukocyte recruitment, ECs exhibit a critical role in the onset as well as the progression of atherosclerosis [14]. Previously, Soe et al. showed that resveratrol blocks LPS-stimulated monocyte binding to ECs by inhibiting ICAM-1 expression [28]. According to another study, MO leaf extract decreases arterial blood pressure by causing small-resistance arteries to relax, mostly by activating the eNOS-NO-sGC pathway [25]. Our previous studies have shown that moringa leaf extract and the active substance 3-HBI possess anti-inflammatory and cancer-preventing capabilities [24,29]. In the current investigation, 3-HBI, fraction 6 of MO leaf extract, and the MO extracts’ activities in TNF-α-activated ECs were all investigated. We found that MO leaf extract, fraction 6, and 3-HBI significantly decreased the production of cytokines and chemokines, including IL-6, IL-8, and MCP-1 (Figure 2), and decreased the cell surface expression of adhesion molecules (ICAM-1, VCAM-1, and PECAM-1). These findings confirm that fraction 6, 3-HBI, and MO leaf extract reduce inflammation in ECs by suppressing cytokine and chemokine secretion as well as adhesion molecule expression. However, a single dose of crude extract, fraction 6 and 3-HBI were tested in this investigation because preliminary experiments showed that low doses and high doses were not significantly different. Moreover, all the crude MO leaf extract, fraction 6, and 3-HBI treatments provided anti-inflammatory activity, but there was no significant difference between extract, fraction, and compound in almost every test. Baker et al. described that plant-derived fatty acids, gamma-linolenic acid (GLA), pinolenic acid (PLA), dihomo-gamma-linolenic acid (DGLA), and eicosatrienoic acid (ETA) decrease the production of ICAM-1, MCP-1, RANTES, and IL-6 in EA.hy926 cells [30]. Another study from Bork et al. demonstrated that a low ratio of linoleic acid (LA) to alpha-linolenic acid (ALA) downregulated the secretion of VEGF, RANTES, ICAM-1, MCP-1, and IL-6 by cytokine-induced EA.hy926 cells [31].

By upregulating adhesion molecules on the endothelium, the cytokine IL-6 regulates the immune system and the vascular inflammatory response [32]. MCP-1 and IL-8 are chemoattractants that facilitate the recruitment and attachment of circulating leukocytes to the sub-endothelial layer [33]. Hence, we further investigated the activity of crude MO leaf extract and 3-HBI on monocyte and endothelial cell interactions and monocyte transmigration. The results showed that the adhesion of calcein-labeled THP-1 cells to EA.hy926 cells was substantially increased by the TNF-α activation of ECs. Pre-treatment of TNFα-activated Ea.hy926 cells with crude extract, fraction 6, or 3-HBI significantly decreased monocytic THP-1 cell adhesion to EA.hy926 cell single-layer cultures in comparison to TNF-α-activated cells alone (*p* ≤ 0.001). In addition, we also analyzed the transmigration of THP-1 monocytes through an EC monolayer by using a transwell transmigration assay. As demonstrated in Figure 5, incubation with crude MO extract, fraction 6, or 3-HBI followed by TNF-α stimulation significantly decreased monocytic THP-1 cell transmigration through the EC monolayer. Before being able to transmigrate through the endothelial monolayer, monocytes have to roll over the monolayer and then firmly stick on it. Stimulated ECs express adhesion molecules that interact with ligands of monocytes. This interaction allows monocytes to roll on the endothelium and transmigrate [34]. Our results highlighted that MO leaf extract blocks monocyte adhesion and transendothelial migration by reducing inflammatory cytokine production and suppressing the generation of adhesion molecules by ECs. These properties of moringa extract and its bioactive ingredient, 3-HBI, may have an impact on a number of clinical conditions, including atherosclerosis and neuroinflammation, which involve monocyte-mediated inflammation. Our findings are important because monocytes have an important function during the early, development, and thrombus formation phases of atherosclerosis and myocardial infarction. Monocytes also contribute to a significant part of the immune system’s inflammatory reaction seen in hypercholesterolemia-related trafficking to inflamed arteries. They are primarily reliant on chemokines, extravasation, subendothelial accumulation, and macrophage differentiation. These cells eventually develop into the harmful cholesterol-rich foam cells that make up atherosclerotic plaques [27,34,35].

Several research projects have indicated that TNF-α-induced NF-κB activation is responsible for ICAM-1, VCAM-1, and PECAM-1, other adhesion molecules, cytokines, and chemokines, being expressed in ECs [26,36]. The primary transcription factor controlling immunological and inflammatory genes connected to atherosclerosis and endothelial dysfunction is NF-κB [37,38]. Chrysin inhibits VCAM-1 expression and monocyte adhesion in bacterial cell wall (LPS)-activated cerebral endothelial cells by regulating NF-κB signaling. Studies have also demonstrated that astragalus polysaccharides reduce ICAM-1 and VCAM-1 expression in TNF-α-exposed human umbilical vein endothelial cells (HUVECs) [39,40]. Our current study showed that ICAM-1 and VCAM-1 expression increased in response to TNF-α, as did NF-κB P65 and IκB phosphorylation, and these increases in ICAM-1, VCAM-1, and NF-κB signaling were significantly attenuated by crude moringa leaf, fraction 6, and 3-HBI pre-treatment in EA.hy926 cells. Thus, these results indicate that reducing adhesion molecule (ICAM-1 and VCAM-1) expression and monocyte transendothelial migration by MO extract and 3-HBI treatment is associated with suppression of the NF-κB pathway. Our findings are consistent with a prior study in which the natural scavenger resveratrol, which is found in red wine and grapes, reduced TNF-α-induced changes in the genes and proteins that are involved in monocyte adhesion in EA.hy926 cells [41]. Similarly, andrographolide attenuates the activation of NF-κB in EA.hy926 cells, which, in turn, reduces TNF-α-increased ICAM-1 expression [16].

Moringa is a popular native food in Thailand and other tropical nations. Almost every component of MO has medicinal and nutritional benefits [42]. The MO leaf in particular has a number of health benefits, such as anti-inflammatory, antioxidant, and anti-hyperglycemic actions that can boost the immune system [19,43]. Numerous in vivo investigations have used MO leaves with no unfavorable results. It could be formulated as a supplement and an agent that prevents the generation of cytokines, chemokines, and adhesion molecules that promote inflammation, halting the onset and progression of vascular diseases including neuroinflammation and atherosclerosis. The main finding from this study was that the repression of the IκB-α/NF-κB signaling cascade by crude MO leaf extract and the bioactive compound 3-HBI can reduce a number of aspects of the inflammatory response in cultured ECs. We present a potential relationship between the inhibiting action of MO leaf extract on the NF-κB signaling cascade and the ability of 3-HBI, the extract’s bioactive component, to prevent vascular endothelial inflammation. Specifically, where the endothelium is concerned, our findings point to MO leaf extract and 3-HBI as promising new medicines that can offer defense against inflammation and inflammatory disorders. However, additional studies are needed to determine the efficacy and mechanism of action of 3-HBI and MO leaf extract in atherosclerosis patient samples; patient-derived xenograft models are useful systems for examining the impacts of combinations of compounds.

## 4. Materials and Methods

### 4.1. The Extraction of MO Leaf and Its Bioactive Compound

Growing of *Moringa oleifera* Lam. in Uthai Thani province of Thailand (farmer registration number 730210-2178-1-1) followed all the applicable guidelines of Thai agricultural standard TAS 9001-2013 (good agricultural practices for food crops), and the leaf collection method and experimental procedure use were certified as such by the Asia medical and agricultural laboratory and research center (AMARC 9001-2556). Leaf dried powder (Lot. No. 5534) originated from the Khaolaor Company, Thailand, as shown in the Supplementary Materials, Figure S1. The procedure for leaf extraction was in accordance with all the relevant guidelines [44]. Briefly, MO leaves were macerated with ethyl acetate (EtOAc) at room temperature. The mixture was filtered using Whatman No.1 filter paper prior to rotary evaporation at 40 °C. As explained previously [24], flash column chromatography (FCC) (Merck, Darmstadt, Germany) was used for separating the fractions from the crude EtOAc extract; hexane, hexane-EtOAc, and EtOAc-methanol (MeOH) were used in a solvent-based system with progressively increasing polarity to perform gradient elution, which produced 15 fractions. Bioassay-guided fractionation was used for profiling and screening of the extracts for bioactive fractions and compounds. The ability of the fractions to suppress the secretion of pro-inflammatory substances was examined by an enzyme-linked immunosorbent assay (ELISA). Fraction no. 6 demonstrated the highest effectiveness and was used for the next round of FCC. A total of 17 sub-fractions were produced, and sub-fraction no. 6.17 was found to be an effective inhibitor of inflammation. This sub-fraction no. 6.17 was separated by silica gel column chromatography, and just a single potent anti-inflammatory portion was revealed (no. 6.17.2) (Supplementary Materials, Figure S2). This was characterized by LC-ESI-QTOF-MS/MS. The moringa extract and fractions were kept in vials at −20 °C and in the dark until being used.

By interrogating the MS and MS/MS data in available databases, a preliminary identification of no. 6.17.2 was made. An active substance, 3-hydroxy-β-ionone (3-HBI), was tentatively identified (Supplementary Materials, Figure S3). 3-HBI was purchased from Santa Cruz Biotechnology Inc. in Dallas, TE, USA, for the experiments described here.

### 4.2. Cell Lines and Tissue Culture Conditions

EA.hy926 endothelial cells (ATCC^®^ CRL 926) were procured from the American Type Culture Collection (ATCC). The cells were cultivated in high-glucose Dulbecco’s Modified Eagle Medium (DMEM) supplemented with 10% fetal bovine serum (FBS), 1% antibiotic-antimycotic (Thermo Fisher Scientific, Inc., New York, NY, USA), and 1% HAT (100 μM hypoxanthine, 0.4 μM aminopterin, and 16 μM thymidine) (Sigma-Aldrich, St. Louis, MO, USA). THP-1 cells were maintained in RPMI 1640 complete medium supplemented with 10% FBS and 1% antibiotic-antimycotic (Thermo Fisher Scientific, New York, NY, USA). All cells were cultured at 37 °C with 5% CO_2_, and the medium was changed every 2–3 days.

### 4.3. Cellular Damage Test

Cellular damage was assessed using an MTT assay. EA.hy926 cells were seeded in a 96-well plate at a density of 1 × 10^4^ cells/well and treated with various concentrations of the extract, fraction 6, and active compound (3-HBI). The cells were then incubated with complete medium with or without TNF-α at a final concentration of 1 ng/mL for 24 h. After incubation, the supernatant was removed, and 50 µL of MTT solution (0.5 mg/mL) in serum-free medium (Invitrogen, Carlsbad, CA, USA) was added to each well. The plates were incubated at 37 °C for 3 h. Subsequently, the MTT reagent was removed, and the formazan crystals were dissolved in 100 µL of DMSO. The absorbance at 590 nm was measured using a microplate reader (PerkinElmer, Inc., Waltham, MA, USA). This method followed a previously established protocol [45]. The concentrations at which 5% inhibition (IC5) and 50% inhibition (IC50) occurred for the crude MO extract and 3-HBI were determined and are shown as sigmoidal curve fits.

### 4.4. Measurement of Pro-Inflammatory Mediator Concentrations

In 24-well plates, EA.hy926 cells were seeded and allowed to grow for 24 h. The cells were then exposed to MO leaf extract (75 µg/mL), fraction 6 (75 µg/mL), or 3-HBI (50 µg/mL) for 24 h, followed by incubation with TNF-α (1 ng/mL) for an additional 24 h. The levels of cytokines and chemokines such as interleukin 6 (IL-6), interleukin 8 (IL-8), and monocyte chemoattractant protein-1 (MCP-1) were assessed using a Bio-Plex multiplex immunoassay system (Bio-Rad Laboratories Inc., Hercules, CA, USA). The manufacturer’s recommendations for solutions and standardization were followed. The plate was read using a Bio-Plex 200 multiplex reader (Bio-Rad Laboratories Inc., Hercules, CA, USA).

### 4.5. Determination of ICAM-1 (CD54) and PECAM-1 (CD31) Expression

The expression of cluster of differentiation (CD) markers was measured through immunostaining using PE-conjugated anti-CD54 and FITC-conjugated anti-CD31 antibodies (BD Biosciences, San Jose, CA, USA). EA.hy926 cells were seeded in 12-well plates and cultured for 24 h at 37 °C. The cells were treated with MO leaf extract (75 µg/mL), fraction 6 (75 µg/mL), or 3-HBI (50 µg/mL) for 24 h before incubation with TNF-α (1 ng/mL) for 6 h. Subsequently, cells from each condition were harvested, washed with PBS, and resuspended in FACS buffer solution. The cells were then stained directly with fluorescence-conjugated antibodies against CD54 or CD31. After an incubation period of 1 h at 4 °C in the dark, the cells were washed twice with PBS. Cell pellets were resuspended in staining buffer, and the fluorescence was measured using an FC 500 Flow Cytometer and CytoFLEX S Flow Cytometer (Beckman Coulter, Inc., Indianapolis, IN, USA). As negative controls, FITC-conjugated mouse IgG1, κ isotype control, and PE-conjugated mouse IgG1, κ isotype control (both from BioLegend, Inc., San Diego, CA, USA), were employed.

### 4.6. THP-1 Monocyte Adhesion to EA.hy926 Endothelium

The adherence of monocytes and endothelial cells was investigated by a Vybrant Cell Adhesion Assay Kit (Thermo Fisher Scientific). EA.hy926 ECs were incubated for 24 h. MO leaf extract (75 µg/mL), fraction 6 (75 µg/mL), or 3-HBI (50 µg/mL) were used to treat cells beforehand for 24 h, and they were then stimulated with TNF-α (1 ng/mL) for 6 h. THP-1 cells were prepared as a cell suspension at 3 × 10^5^ cells/mL. Calcein was added to the THP-1 cell suspensions in serum-free media, and monocytes were cultured at 37 °C for 30 min. THP-1 monocytes were washed 3 times with serum-free medium. Calcein-labeled monocytes were co-cultured with treated EA.hy926 cells at 37 °C for 1 h. Non-adherent monocytes were discarded by gentle washing. A total of 100 µL of PBS was added to each well, and the fluorescence was detected by a fluorescence plate reader at 480 nm/520 nm. THP-1 monocyte adhesion was calculated as a proportion of the TNF-α-activated monocytes (without treatment with extract, sub-fraction of bioactive compound). To calculate the percentage, the O.D. of the MO extract, fraction 6, or 3-HBI was divided by the O.D. of the TNF-α-treated cells and then multiplied by 100. A Nikon H600l microscope (Nikon corporation, Minato-ku, Tokyo, Japan) was used to capture pictures of fluorescence-labeled cells attached to the endothelium.

### 4.7. Monocyte Transmigration Assay

THP-1 cell migration through EC monolayers was investigated by using a leukocyte transendothelial migration assay. EA.hy926 cells (5 × 10^4^) in 400 µL of media were introduced to each insert in a 24-well plate containing 500 µL of medium. Cells were cultured for 72 h until the endothelial cells formed a monolayer. The cells were pre-treated with MO leaf extract, fraction 6, or 3-HBI and stimulated with TNF-α as described above. The culture medium was carefully discarded, and the insert was moved to a different well containing 500 µL of DMEM including N-formyl-methionyl-leucyl-phenylalanine (FMLP; 20 nM) as a chemoattractant. A fluorescence-labeled THP-1 cell suspension was prepared and added to each insert and cultured at 37 °C for 12 h. The media beneath the container containing the migrating cells was removed to a 96-well plate suitable for fluorescence measurement on a fluorescence plate reader at 480 nm/520 nm.

### 4.8. Western Blotting

Total protein was extracted from the EA.hy926 cells using cooling RIPA lysis buffer (Bio Basic Inc., Amherst, NY, USA) supplemented with Halt Protease/Phosphatase Inhibitor Cocktails (Thermo Fisher Scientific, NY, USA) and then centrifuged at 12,000 rpm for 15 min at 4 °C. The total protein concentration was quantified using a Bradford Coomassie-binding colorimetric assay. The samples were mixed with the same amount of 4 × Laemmli loading buffer and warmed at 95 °C for 5 min, during which the proteins were denatured. The proteins were loaded onto 10% or 12% SDS-polyacrylamide gels alongside a pre-stained marker protein ladder (6.5–270 kDa) and subjected to polyacrylamide gel electrophoresis (PAGE). The gels were initially run at 100 V for 10 min to align the proteins, followed by an increase in voltage to 150 V for 1–1.5 h. The proteins were then transferred from the gels to a polyvinylidene fluoride membrane (Bio-Rad Laboratories Inc., Hercules, CA, USA). Using a solution containing 5% bovine serum albumin (Capricorn Scientific GmbH, Ebsdorfergrund, Germany) in Tris-buffered saline with Tween 20 (TBST), the membranes were blocked for 2 h at room temperature. Subsequently, the membrane was probed with primary antibodies specific to β-actin (cat no, PA1-183), phospho-NFκB p65 (Ser536) (cat no. MA5-15160), total NF-κB P65 (cat no.51-0500), total IκBα (cat no.710128) (Thermo Fisher Scientific), phospho-IκB-α (Ser32) (cat no. 2859) (Cell Signaling Technology, Beverly, MA, USA), ICAM-1 (cat no. 109361), and VCAM-1 (cat no. 134047) (Abcam Inc., Waltham, MA, USA) on a rotator overnight at 4 °C. After washing with TBST, the polyvinylidene fluoride membrane was incubated with horseradish peroxidase-conjugated goat anti-rabbit IgG (H + L) secondary antibody (cat no.31460) (Thermo Fisher Scientific, NY, USA) at room temperature for 1 h. Chemiluminescence substrate was applied to the membrane for 5 min, and the signal was detected using a ChemiDoc XRS+ Imaging System (Bio-Rad Laboratories Inc., Hercules, CA, USA). The chemiluminescence signal from the blotted vinyl was analyzed using the Image Studio Lite software version 5.2 (LI-COR Corporation, Lincoln, NE, USA).

### 4.9. Statistical Analysis

To ensure accurate results, every experimental condition was tested in triplicate. Data analysis was performed with the GraphPad Prism software version 8 using one-way ANOVA and the Bonferroni multiple comparisons test. All statistical analyses employed a 95% confidence interval (*p* = 0.05).

## 5. Conclusions

In conclusion, the findings of this in vitro study showed that the production of IL-6, IL-8, and MCP-1 and the upregulation of ICAM-1, VCAM-1, and PECAM-1 in ECs were triggered by TNF-α. Furthermore, the adhesion of monocytic THP-1 cells to EA.hy926 cells and the transendothelial migration of THP-1 monocytes were shown to significantly increase with the TNF-α stimulation of endothelial cells. All these consequences of TNF-α stimulation can be reversed by pre-treatment with MO leaf extract and 3-HBI, which appears to be due to inhibition of the IκB-α/NF-κB signaling pathway. These findings suggest that MO leaves are potential foods and dietary supplements for reducing the development of vascular disorders, including atherosclerosis and neuroinflammation.

## Data Availability

Data are contained within the article and Supplementary Materials.

## Supplementary Materials

The following supporting information can be downloaded at: https://www.mdpi.com/article/10.3390/molecules29245873/s1, Figure S1: Certificate of analysis (COA) of moringa powder; Figure S2: Bio-guided fractionation assay of crude moringa extract; Figure S3: LC-ESI-QTOF-MS/MS analysis; Figure S4: Sodium dodecyl sulfate–polyacrylamide gel electrophoresis (SDS-PAGE); Figure S5: Original blots of beta-actin and protein ladder; Figure S6: Original blots of VCAM-1 and protein ladder; Figure S7: Original blots of ICAM-1 and protein ladder; Figure S8: Original blots of pNF-kB P65 and protein ladder; Figure S9: Original blots of total NF-kB P65 and protein ladder; Figure S10: Original blots of p-IkB-α and protein ladder; Figure S11: Original blots of total IκB-α and protein ladder.
