# Supplementary material for: Anti-Inflammatory Potential of 3-Hydroxy-β-Ionone from Moringa oleifera: Decreased Transendothelial Migration of Monocytes Through an Inflamed Human Endothelial Cell Monolayer by Inhibiting the IκB-α/NF-κB Signaling Pathway
Source: Molecules. 2024 Dec 12;29(24):5873. doi: 10.3390/molecules29245873 (PMC11678794; doi:10.3390/molecules29245873)
Supplement: Supplementary file 1 [file molecules-29-05873-s001.zip › molecules-3306211-supplementary.pdf]

Additional file

Anti-inflammatory potential of 3-Hydroxy-β-Ionone from *Moringa oleifera* : decrease transendothelial migration of monocytes in Inflamed Human Endothelial cells by Inhibiting IKB-α/NF-κB signaling pathway.

Thitiya Luetragoon[1,2], Yordhathai Thongsri[2], Pachuen Potup[2], Philip C. Calder[3] and Kanchana Usuwanthim[2]*

*** Correspondence:** Kanchana Usuwanthim: Kanchanau@nu.ac.th

KLO

CERTIFICATE OF ANALYSIS

**Product Name**	:	Moringa oleifera dried Powder
**Lot No.**	:	5534
**Manufacture Date**	:	05/04/2018
**Expiry Date**	:	05/04/2020

Item	Specification	Results
**Physical Control**		
Appearance	Greenish powder	Greenish powder
Odor & Taste	Characteristic	Characteristic
Loss on Drying	10 % Max	4.84%
Bulk Density	0.400 - 0.600 g./ml.	0.400 g./ml.
**Chemical Control**		
Arsenic (As)	< 2 ppm	< 2 ppm
Lead (Pb)	< 1 ppm	< 1 ppm
Cadmium (Cd)	< 0.3 ppm	< 0.3 ppm
**Microbiological Control**		
Total plate count	< 10,000 cfu/g	< 10,000 cfu/g
Yeast & Mold	< 500 cfu/g	< 500 cfu/g
Staphylococcus aureus	Absent / 1 g	Absent / 1 g
Clostridium spp.	Absent / 10 g	Absent / 10 g
Salmonella spp.	Absent / 10 g	Absent / 10 g

Conclusion : ☑ **Approved** ☐ **Rejected**

Analyst Sujitta S.
Q.C. Analyst
Date 07-04-18.

Checked by Wimolrat C.
Head of Quality Control
Date 07-04-18

PIC/s GMP | HALAL
Manufacture of : Herbal Medicine, Health Food, Instant Powder Drink, Standardized Herbal Extracts.

บริษัท ขาวละออเภสัช จำกัด 146/22 ถ.สุขสวัสดิ์ อ.พระสมุทรเจดีย์ จ.สมุทรปราการ 10290
KHAOLAOR LABORATORIES CO.,LTD. 146/22 Suksawad Road, Samutprakan [illegible]
Tel. (66) 2819 7991-5, (66) 2425 1188 Fax (66) 2425 8332
http://www.khaolaor.com E-mail: info@khaolaor.com

F-QC-002
REV.01

Figure S1. Certificate of analysis (COA) of Moringa powder.

Moringa oleifera Lam. (MO) leaf dried powder (Lot. No.5534) were obtained from Khaolaor Laboratories Co., Ltd. (Samut Prakan, Thailand). The contamination of MO powder was analyzed by the company. Arsenic (As), lead (Pb), and cadmium (Cd) were found less than 2 ppm, 1 ppm, and 0.3 ppm, respectively. Yeast and mold < 500 colony-forming unit (cfu)/g was found in MO powder. Bacteria including *Salmonella* spp., *Clostridium* spp., and *Staphylococcus aureus* were not found in MO powder.

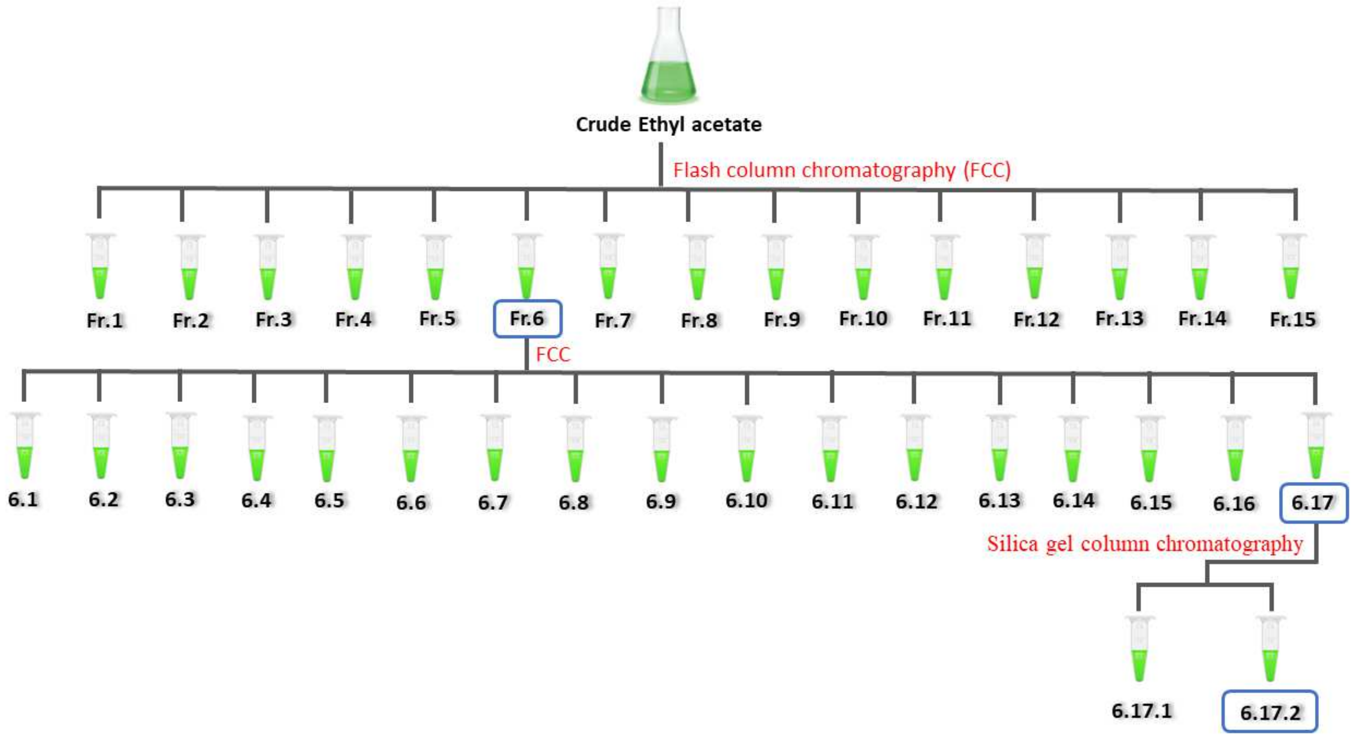

Figure S2. Bio-guided fractionation assay of Crude Moringa extract.

Moringa leaf were macerated with ethyl acetate (EtOAc) at room temperature for 2 days. Extract was filtered through Whatman No.1 filter paper before rotary evaporation at 40 °C. Then, fractions were separated from the crude EtOAc extract using flash column chromatography (FCC) (Merck, Darmstadt, Germany); gradient elution was performed using a solvent system with gradually increasing polarity including hexane, hexane-EtOAc and EtOAc-methanol (MeOH). All fractions were subjected to screening for the inhibition of pro-inflammatory cytokine production. The fraction no. 6 was strongest activity then subjected for secondary fractionation by FCC. There were 17 sub-fractions (Fr.6.1–Fr.6.17), with only sub-fraction no.6.17 showing active anti-inflammation. Then it was partitioned by silica gel column chromatography, the result showed only one active anti-inflammation fraction (no.6.17.2). Therefore, it was subjected to compound identification by LC-ESI-QTOF-MS/MS.

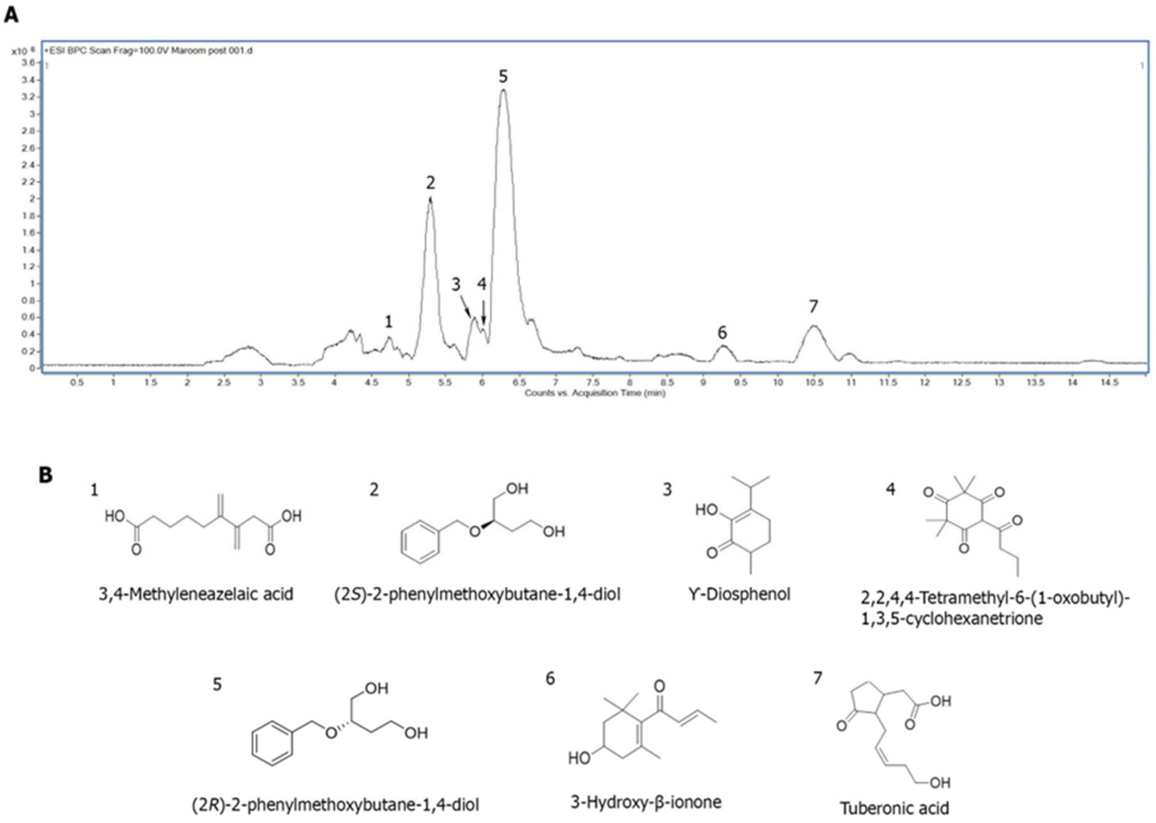

Figure S3. LC-ESI-QTOF-MS/MS analysis

Total ion chromatogram (TIC) of subfraction 6.17.2 obtained from LC-ESI-QTOF-MS/MS (A). Peak identification was performed by comparison of the retention times and mass spectra. The tentative identified compounds were analyzed by published database and library search including 4-Methyleneazelaic acid, (2S)-2-phenylmethoxybutane-1,4-diol isomer 1, gamma-Diosphenol, 6-butanoyl-2,2,4,4-tetramethylcyclohexane-1,3,5-trione, (2S)-2-phenylmethoxybutane-1,4-diol isomer 2, 3-Hydroxy-beta-ionone, and Tuberonic acid (B). Peak identification was performed by comparison of the retention times and mass spectra. The tentative identified compounds were analyzed by published database and library search such as Chemspider (http://www.chemspider.com), Massbank (http://www.massbank.eu) and Human Metabolome Database (http://www.hmdb.ca). The molecular formula was created by Agilent MassHunter Qualitative Analysis Software B.06.0.

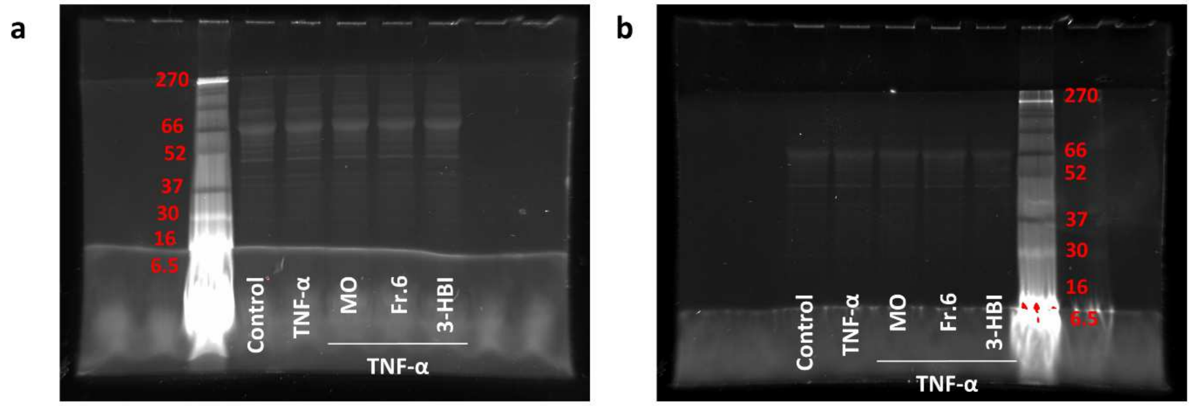

Figure S4. Sodium dodecyl sulfate-polyacrylamide gel electrophoresis (SDS-PAGE).

These images were acquired by ChemiDoc XRS+ Imaging System (Bio-Rad Laboratories Inc., Hercules, CA, USA). (a, b) Sample were load into 10-12% SDS-polyacrylamide gels alongside a blultra prestained protein ladder (6.5-270 kDa) (Bio-Helix Co., LTD). Gels were run at 100 V for 10 minutes followed by increasing the voltage to 150 V for 1 – 1.5 hours.

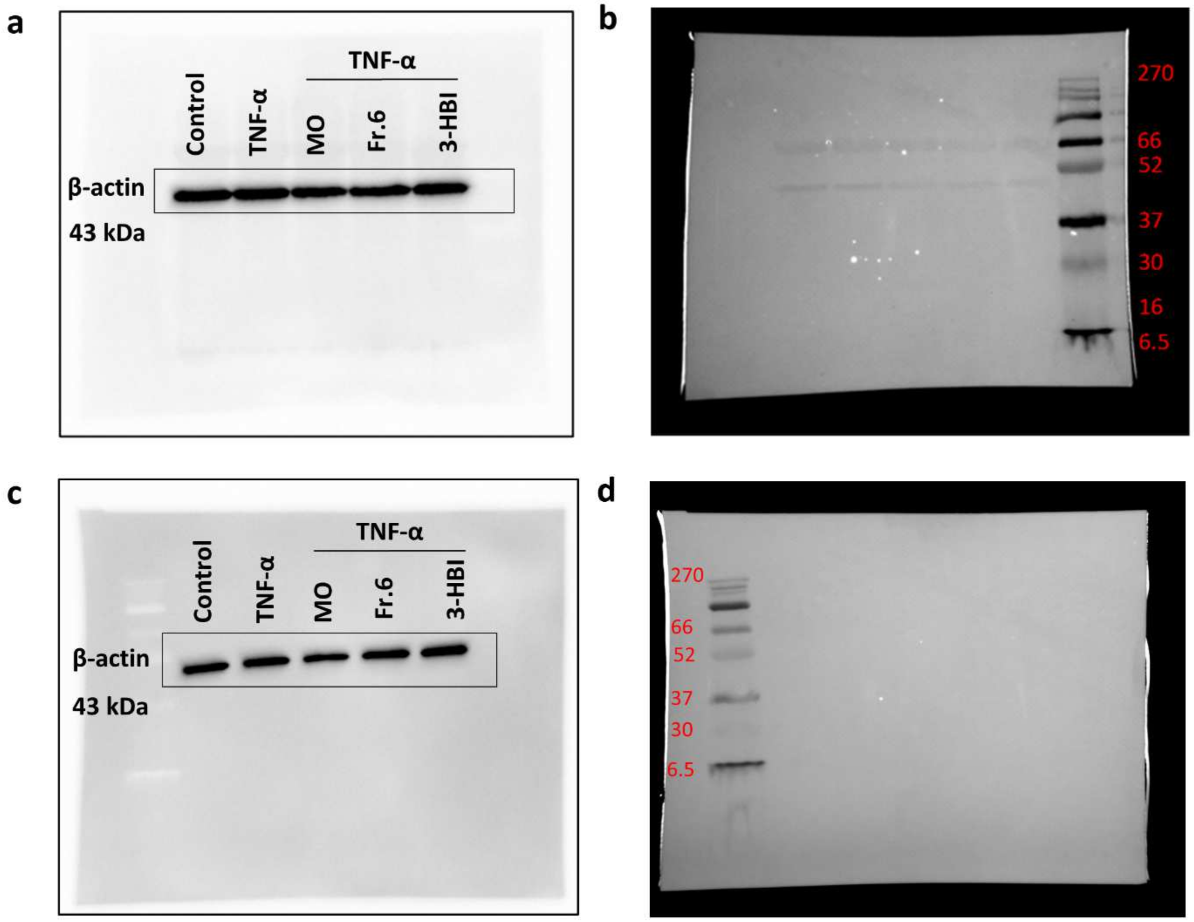

Figure S5. Original blots of Beta-actin and protein ladder.

The protein markers were obtained from Bio-Helix Co., LTD. Blultra prestained protein ladder Cat.no. PMB01-0500. These images were acquired by ChemiDoc XRS+ Imaging System (Bio-Rad Laboratories Inc., Hercules, CA, USA). (a, c) Band intensity of total protein levels of Beta-actin. It was used as loading control. (b, d) Band of Protein marker (6.5-270 kDa).

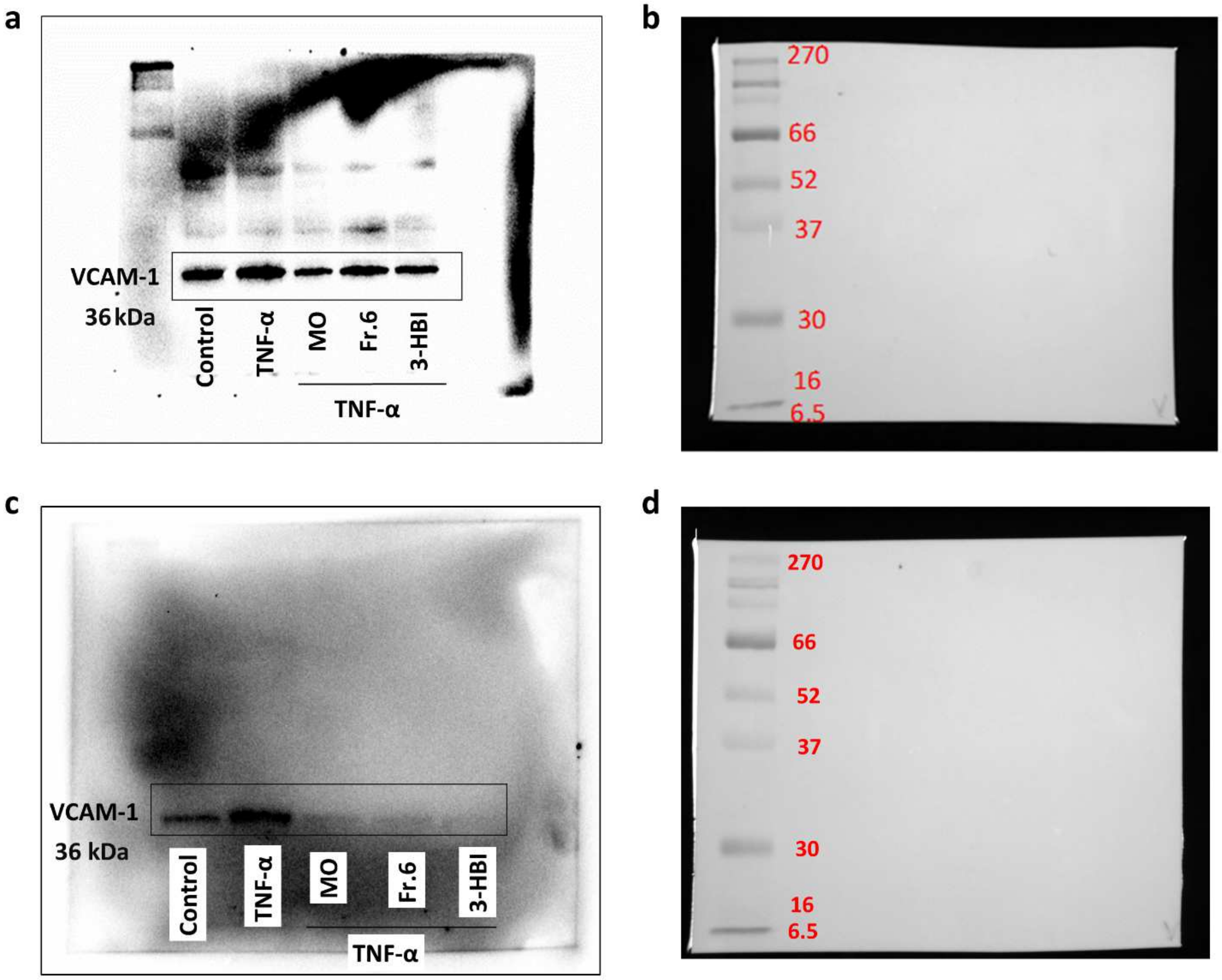

Figure S6. Original blots of VCAM-1 and protein ladder.

The protein markers were obtained from Bio-Helix Co., LTD. Blultra prestained protein ladder Cat.no. PMB01-0500. These images were acquired by ChemiDoc XRS+ Imaging System (Bio-Rad Laboratories Inc., Hercules, CA, USA). (a, c) Band intensity of total protein levels of VCAM-1. (b, d) Band of Protein marker (6.5-270 kDa).

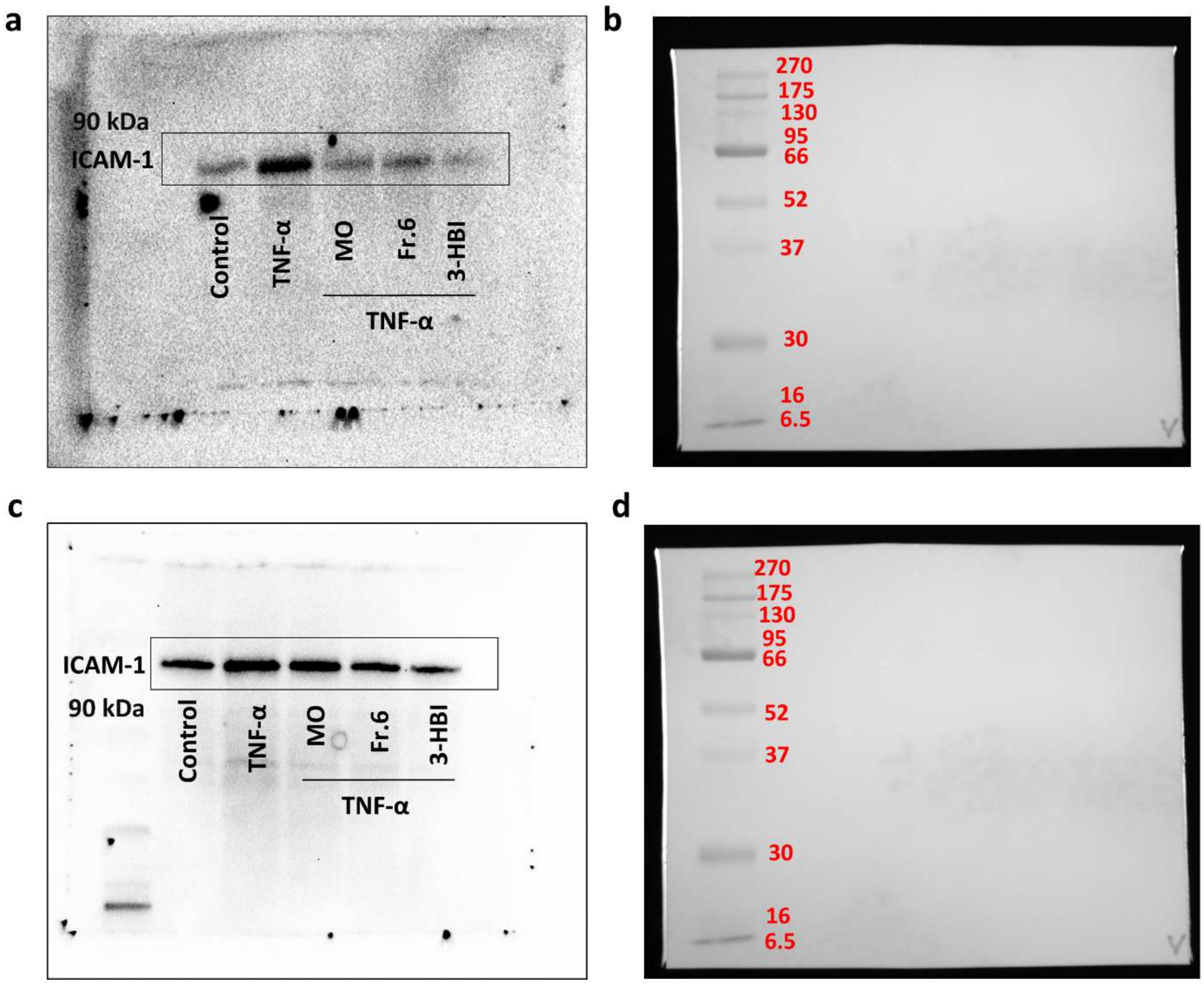

Figure S7. Original blots of ICAM-1 and protein ladder.

The protein markers were obtained from Bio-Helix Co., LTD. Blultra prestained protein ladder Cat.no. PMB01-0500. These images were acquired by ChemiDoc XRS+ Imaging System (Bio-Rad Laboratories Inc., Hercules, CA, USA). (a, c) Band intensity of total protein levels of ICAM-1. (b, d) Band of Protein marker (6.5-270 kDa).

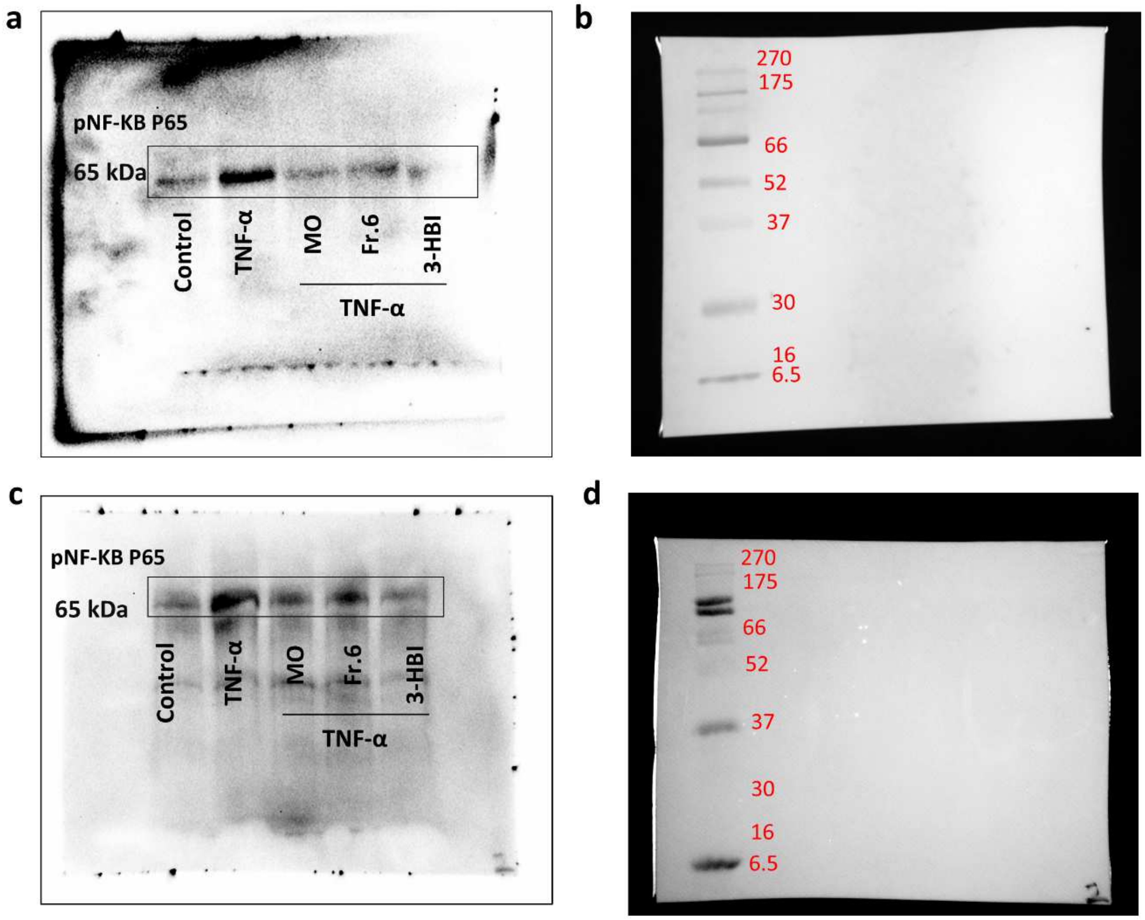

Figure S8. Original blots of pNF-KB P65 and protein ladder.

The protein markers were obtained from Bio-Helix Co., LTD. Blultra prestained protein ladder Cat.no. PMB01-0500. These images were acquired by ChemiDoc XRS+ Imaging System (Bio-Rad Laboratories Inc., Hercules, CA, USA). (a, c) Band intensity of total protein levels of pNF-KB P65. (b, d) Band of Protein marker (6.5-270 kDa).

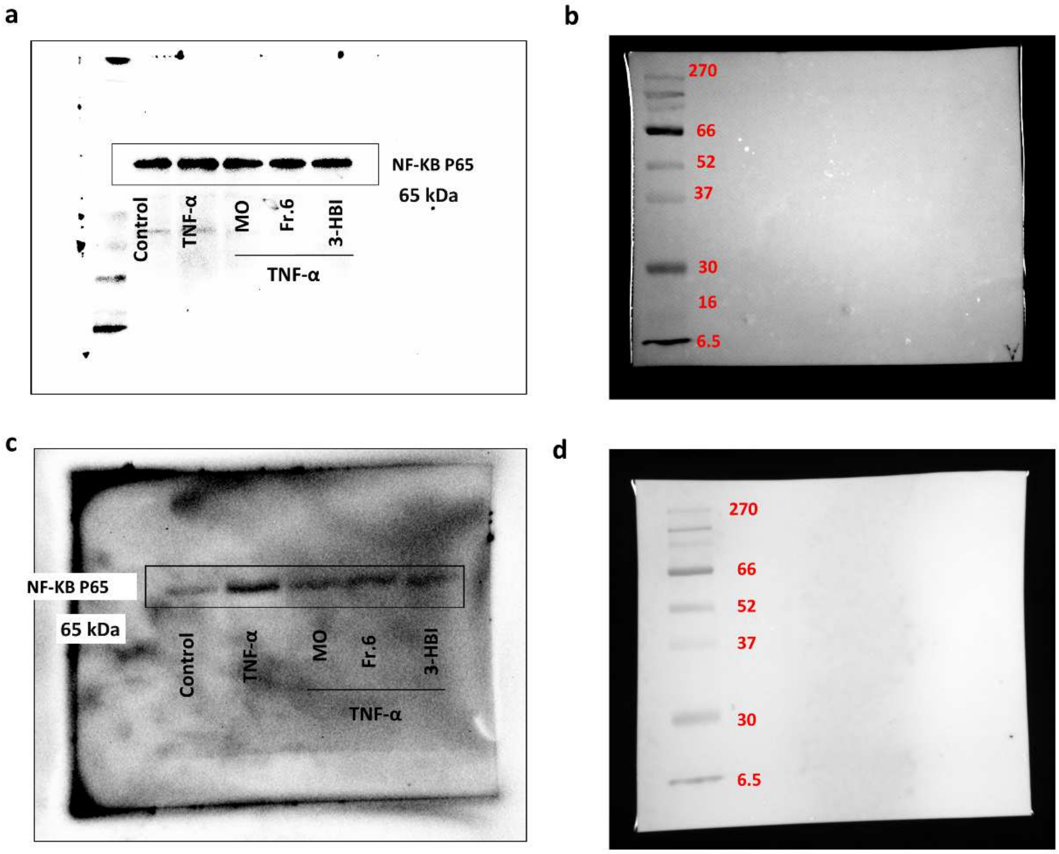

Figure S9. Original blots of total NF-KB P65 and protein ladder.

The protein markers were obtained from Bio-Helix Co., LTD. Blultra prestained protein ladder Cat.no. PMB01-0500. These images were acquired by ChemiDoc XRS+ Imaging System (Bio-Rad Laboratories Inc., Hercules, CA, USA). (a, c) Band intensity of total protein levels of pNF-KB P65. (b, d) Band of Protein marker (6.5-270 kDa).

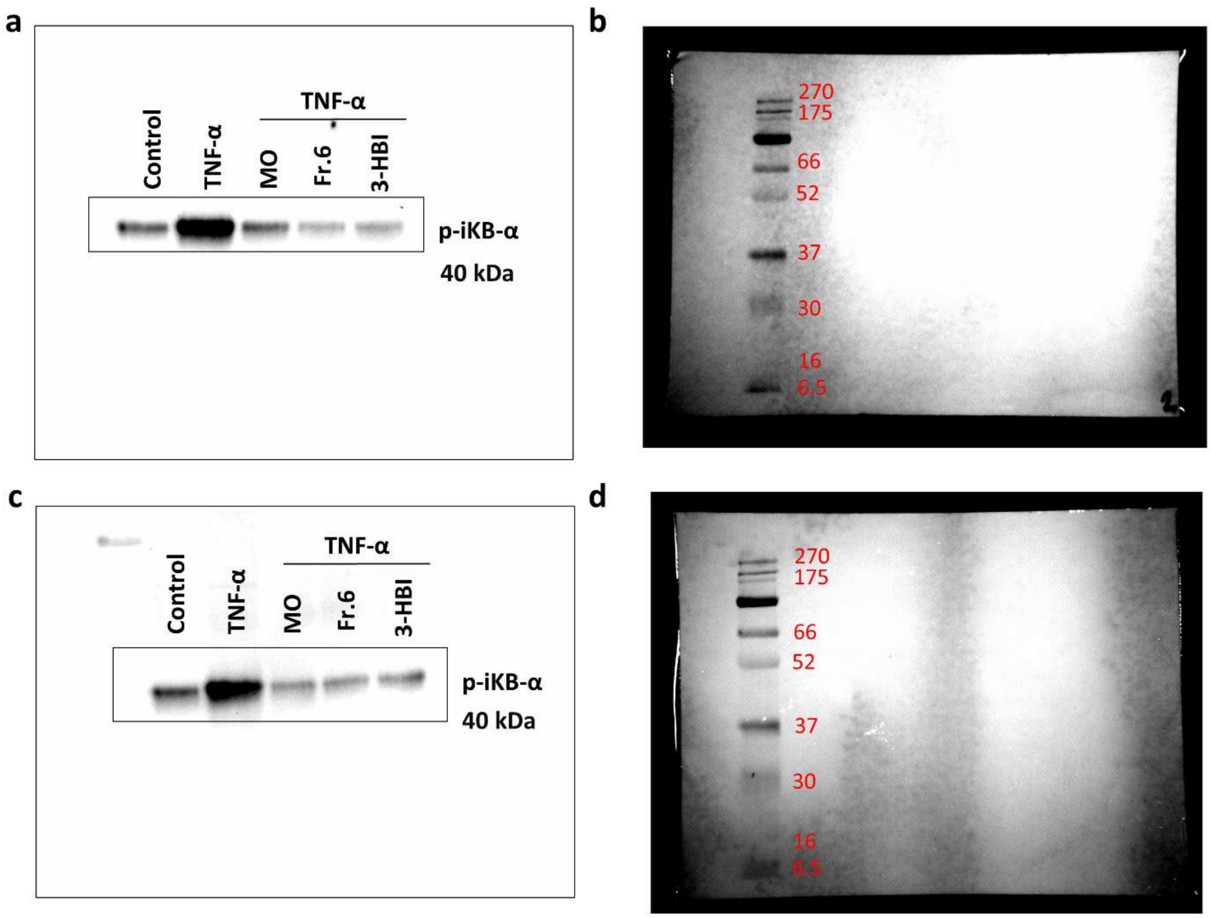

Figure S10. Original blots of p-iKB-α and protein ladder.

The protein markers were obtained from Bio-Helix Co., LTD. Blultra prestained protein ladder Cat.no. PMB01-0500. These images were acquired by ChemiDoc XRS+ Imaging System (Bio-Rad Laboratories Inc., Hercules, CA, USA). (a, c) Band intensity of total protein levels of p-iKB-α. (b, d) Band of Protein marker (6.5-270 kDa).

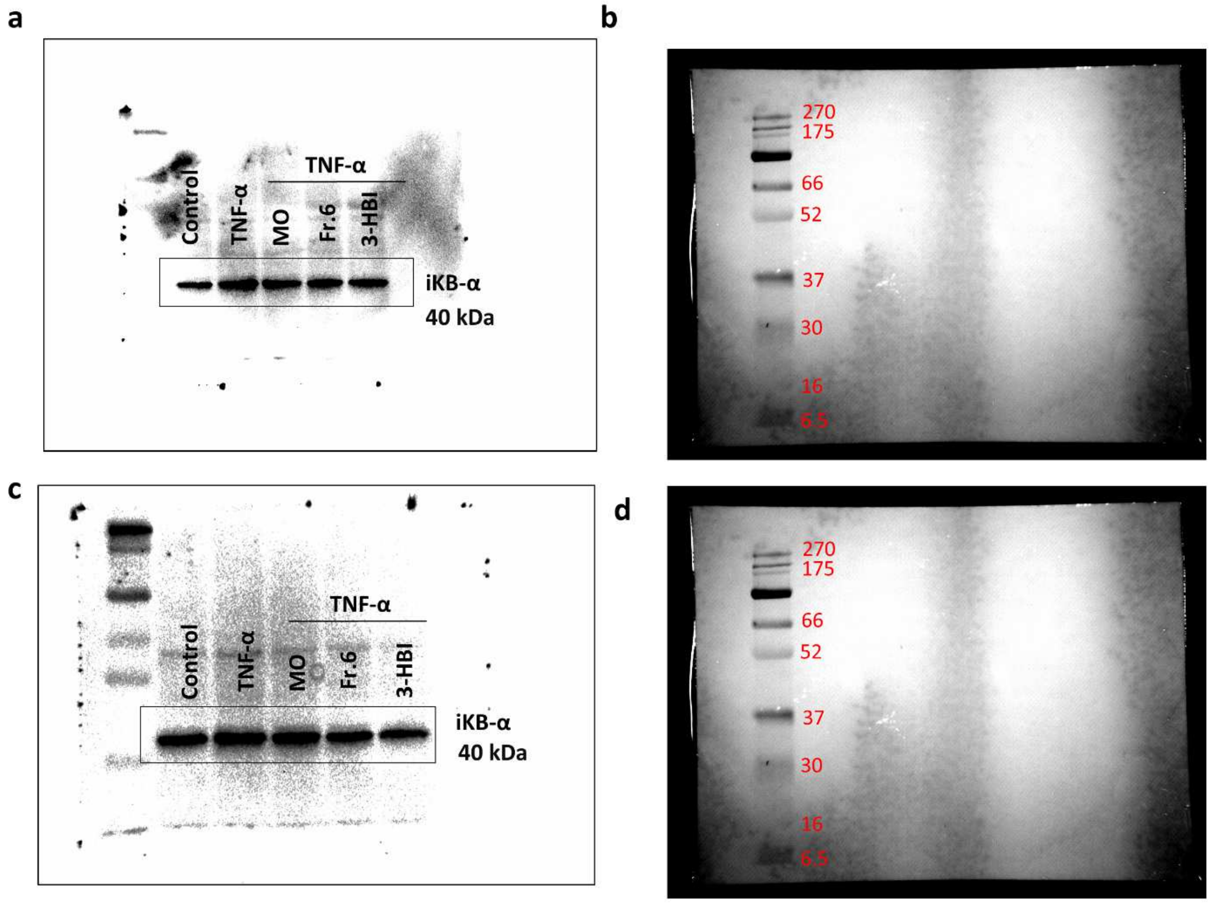

Figure S11. Original blots of total iKB-α and protein ladder.

The protein markers were obtained from Bio-Helix Co., LTD. Blultra prestained protein ladder Cat.no. PMB01-0500. These images were acquired by ChemiDoc XRS+ Imaging System (Bio-Rad Laboratories Inc., Hercules, CA, USA). (a, c) Band intensity of total protein levels of p-iKB-α. (b, d) Band of Protein marker (6.5-270 kDa).
